# Prevalence, Risk Factors for Exposure, and Socio-Economic Impact of Peste Des Petits Ruminants in Karenga District, Karamoja Region, Uganda

**DOI:** 10.3390/pathogens11010054

**Published:** 2022-01-02

**Authors:** Claire Julie Akwongo, Melvyn Quan, Charles Byaruhanga

**Affiliations:** 1 Department of Biomedical Sciences, Institute of Tropical Medicine, Nationalestraat 115, 2000 Antwerp, Belgium; 2 Vectors and Vector-Borne Diseases Research Programme, Department of Veterinary Tropical Diseases, Faculty of Veterinary Science, University of Pretoria, Private Bag X04, Onderstepoort 0110, Pretoria 0002, South Africa; melvyn.quan@up.ac.za (M.Q.); cbyaruhanga27@yahoo.com (C.B.)

**Keywords:** goat, sheep, surveillance, pastoralist, morbillivirus, transboundary animal disease

## Abstract

Peste des petits ruminants (PPR), a disease caused by small ruminant morbillivirus (SRM), is highly contagious with high morbidity and mortality. Controlling PPR requires a proper understanding of the epidemiological dynamics and impact of the disease in a range of geographical areas and management systems. Karenga district, located in the pastoral region of Karamoja in northeastern Uganda, and in the vicinity of Kidepo Valley National Park, is characterised by free cross-border (South Sudan and Kenya) livestock trade, communal grazing, and transhumance. This study was conducted from November through December 2020 to determine the seroprevalence of anti-SRM antibodies, the risk factors associated with the occurrence, and the socio-economic impact of PPR in Karenga. A total of 22 *kraals* were randomly selected from all administrative units, and 684 small ruminants (sheep = 115, goats = 569) were selected for serum collection using systematic random sampling. Exposure to SRM was determined using a competitive enzyme-linked immunosorbent assay. The overall true seroprevalence of SRM antibodies was high, 51.4 (95% confidence interval [CI] 45–52.6). Multivariate logistic regression for risk factors showed that seroprevalence varied significantly by location (26.8% to 87.8%, odds ratio (OR) ≤ 14.5). The odds of exposure to SRM were higher in sheep (73.9%) than in goats (43.8%) (OR = 1.7, *p* = 0.08), and seropositivity was higher in animals greater than two years old (65.5%; OR = 11.1, *p* < 0.001), or those one to two years old (24.7%; OR = 1.6, *p* = 0.2), compared to small ruminants less than one year old (16.1%). Using participatory epidemiology approaches (semi-structured interviews, clinical examinations, pairwise ranking, proportional piling, impact matrix scoring) with 15 key informants and 22 focus groups of pastoralists, PPR was the second most important small ruminant disease: relative morbidity 14%, relative mortality 9%, and case fatality rate 78%, and impacted productivity mainly in terms of treatment costs, mortality, marketability, and conflicts. These findings provide evidence to support the implementation of disease surveillance and control strategies to mitigate the impact of PPR in Karamoja and other pastoral areas in eastern Africa.

## 1. Introduction

Peste des petits ruminants (PPR), also known as goat plague or pseudo rinderpest, is a highly contagious transboundary disease of mostly small ruminants, caused by small ruminant morbillivirus (SRM) in the family *Paramyxoviridae*. The disease is characterised by high morbidity (80–90%) and mortality (50–80%) rates [1]. The virus is lymphotropic and epitheliotropic and affects the respiratory and digestive systems.

A significant association of PPR with pastoral production has been reported, and this can be attributed to movements for trade and grazing that may increase the contact of susceptible animals with infected animals [2]. The effects of PPR directly impact the livelihoods of the pastoral communities by threatening food security, retarding current efforts towards sustainable development, and draining the already-limited veterinary resources [3,4]. Few studies have examined the social and economic consequences of PPR in pastoral areas [5,6,7,8] and there is scanty information about the impact in the Karamoja region, Uganda.

The Karamoja region is predominantly a pastoral area in Uganda and the least developed region. This is where the first outbreak of PPR was reported in the country (July 2017). The unique climatic (semi-arid) conditions, intense cross-border interactions of livestock, and poor veterinary services are undoubtedly a strong justification for more effort in transboundary diseases research. In Karamoja, livestock is an important livelihood asset and the Karimojong pastoralists regard livestock as a “moving bank”, a source of nutrition (meat and milk), and quick cash income for households. The goat and sheep populations in Karamoja were estimated to be 16.4% (n = 2,025,293) and 49.4% (n = 1,685,502) of the national population [9]. However, this small ruminant population in the region is threatened by livestock diseases including PPR. The overall prevalence of PPR among goats in four districts of Karamoja (Moroto, Nakapiripirit, Kotido, and Abim districts) was found to be 57.6% [10], suggesting a relatively high exposure of the animals to SRM. In another study in selected sub-counties in seven districts of Karamoja, the seroprevalence of anti-SRM antibodies in small ruminants was 51.4% in the north (Kaabong, Kotido, and Moroto) and 40.7% in the south (Abim, Napak, Nakapiripirit, and Amudat) [11].

A laboratory and participatory-based assessment of PPR patterns in seven districts of Karamoja (Kaabong, Kotido, Abim, Moroto, Napak, Nakapiripirit, and Amudat) revealed two transmission hotspots that were closely linked to outbreaks from neighbouring countries, especially Kenya, and attributed to cross-border movement of livestock. Within small areas in Karamoja, the foci of infection were attributed to separate systems of management [11]. Karenga district, located in the northern part of the Karamoja region, constitutes a unique PPR epidemiological situation. The district is located in the vicinity of Kidepo Valley National Park and is bordered by South Sudan in the north. The free cross-border trade in goats and sheep, communal grazing of animals, and informal livestock marketing are interesting characteristics of the small ruminant production chain in this northern part of the Karamoja region.

This study aimed to obtain a better understanding of the epidemiological situation of PPR in the Karenga district, as well as the impact of the disease on the livelihoods of small ruminant holders. This can contribute to the realization of the objectives of the PPR Global Eradication Programme (GEP), which emphasises control and eradication efforts by regions/zones of similar epidemiological patterns through improved knowledge on the epidemiology of the virus, enhanced monitoring for exposure as well as assessment of socio-economic impact in a range of geographic areas and management systems.

## 2. Materials and Methods

### 2.1. Study Area

**Figure 1:**
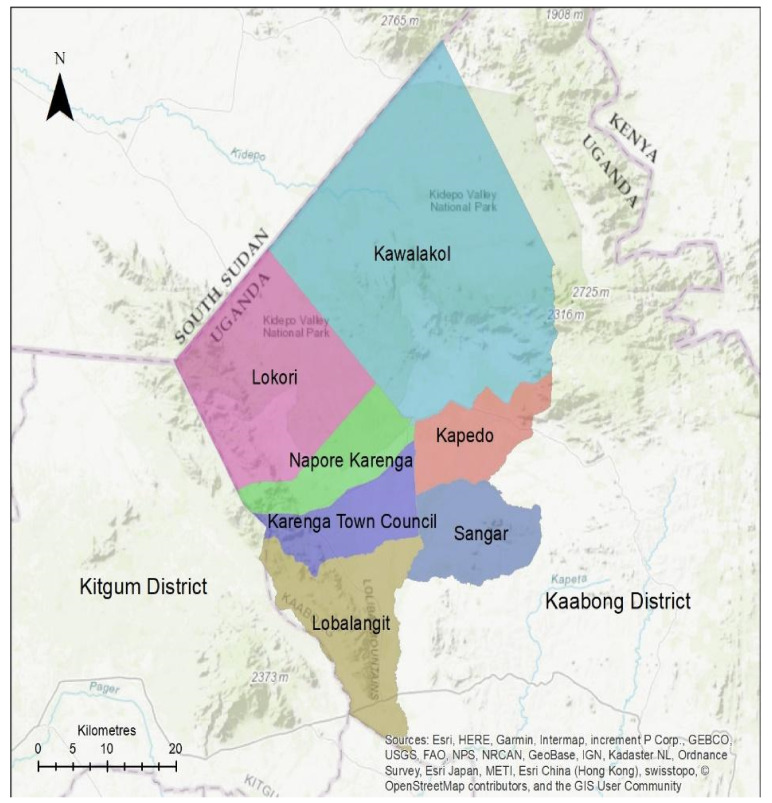
Map of Karenga district in Karamoja region (northeastern Uganda) showing the study sites and neighbouring districts and countries.

The study was conducted in the Karenga district, located in the Karamoja region in north-eastern Uganda. The district is divided into six sub-counties: Sangar, Kapedo, Kawalakol, Lobalangit, Lokori, Karenga, and one town council: Karenga (Figure 1) It is bordered by South Sudan to the northwest, Kaabong district in the east and south, Kotido district in the south and Kitgum district in the west. The district contains the Kidepo Valley National Park, which covers approximately 1442 square kilometres and expands beyond Uganda into South Sudan. Karenga, as other parts of the Karamoja region, experiences a dry savannah semi-arid climate characterized by an intense dry season from November to March each year, with whirlwinds and dust storms, heavy rains from April to June, and light rains from July to October. The rainfall is erratic and averages about 519 mm per annum. The daily temperatures range from 20 °C to 32 °C; relative humidity can reach 60% between June and July [12,13].

### 2.2. Determination of Prevalence of PPR

#### 2.2.1. Study Design and Sample Size

A cross-sectional design was employed and consisted of a two-stage cluster sampling strategy, in which the primary sampling units, small ruminant herds (*kraals*), were selected by simple random sampling from the district, followed by a systematic random selection of individual animals from within each *kraal*. A *kraal* in the Karimojong context is a group of herds that share grazing grounds and management and are found in the same settlement enclosure (*manyatta*).

The sample size was calculated using a formula derived from Dohoo and Stryhn [14]:

(1) $$n=Z_{\alpha }^{2}p\left(1-p\right)/d^{2}$$

where n = sample size, Z = statistic for level of confidence = (1 − α), p = *a priori* estimate of the prevalence and d = precision of the estimate. A sample size of 384 was calculated using a level of confidence of 95% (Z = 1.96), an estimated prevalence of 51.4% from a previous study in Karamoja by Nkamwesiga, Coffin-Schmitt, Ochwo, Mwiine, Palopoli, Ndekezi, Isingoma, Nantima, Nsamba and Adiba [11] and a precision of 5% (0.05).

Given that cluster sampling was employed, the sample size was adjusted by multiplying it with the design effect (DE), to take into consideration the expected dependence of exposure in each cluster (*kraal*). The design effect was determined using a formula described by Killip, et al. [15]:

DE = 1 + ρ (n − 1)(2)

where ρ is the intracluster correlation coefficient, estimated at 0.02 [16], for being seropositive for SRM in a herd and n is the average small ruminant herd size (40). The calculated DE was 1.78, and the effective sample size obtained was 684 small ruminants.

The average number of small ruminants to be sampled within each kraal (n) was estimated to be 31 following a formula described by Elbers, et al. [17]:

(3) $$n=\sqrt{C1/C2\times \left(1-\varrho \right)/\varrho }$$

where C1 is the cost of sampling a *kraal*, C2 is the cost of sampling an animal within a *kraal* and ρ is the intracluster (intra-*kraal*) correlation coefficient (0.02). Given the estimated distance between *kraals*, C1 was estimated to be 20 times higher than C2. The number of *kraals* to be sampled (22) in the seven administrative units was calculated by dividing the total sample size of animals (684) by the sampling size per *kraal* (31).

#### 2.2.2. Clinical Examination and Blood Samples

Before blood collection from the selected goats and sheep, data were collected on the source of each animal, length of stay in the herd, vaccination status and whether or not it had ever been taken to market and returned. Information on the age (using dentition), sex, sub-county, herd/flock size were also recorded. Animals were examined clinically, and any signs related to PPR were noted. Animals less than five months of age were not sampled as maternal antibodies that could be present in these samples could confound the results. Blood samples (3 to 5 mL) were collected from the jugular vein of each animal into labelled vacutainers without anti-coagulant and transported on ice to the Karenga district veterinary department where they were stored at 4 °C overnight, followed by the separation of the sera. The serum samples were aliquoted into 2 mL cryotubes and then transported on ice to the Central Diagnostic Laboratory (CDL), College of Veterinary Medicine, Animal Resources and Biosecurity (CoVAB), Makerere University, Kampala, and stored at −20 °C until further analysis.

#### 2.2.3. Competitive Enzyme-Linked Immunosorbent (cELISA) Assay

The serum samples were tested using a commercial cELISA platform, ID Screen^®^ PPR Competition (IDvet Innovative Diagnostics, Grabels, France) following the manufacturer’s OIE-recommended protocol. The specificity and sensitivity of the cELISA assay are 99.4% 94.5%, respectively [18]. The optical density (OD) was recorded at 450 nm using the Asys UVM 340 Microplate Reader (Biochrom Ltd., Cambridge, UK). The cut-off points were calculated as percentage inhibition (S/N) from the optical densities (OD), as described by Libeau, Préhaud, Lancelot, Colas, Guerre, Bishop and Diallo [18]:

S/N (%) = (OD_test sample_/OD_blank_) × 100 (4)

where OD_blank_ is the OD of the negative control. Samples that showed S/N values of less than or equal to 50% were considered positive, those with S/N greater than 50% were considered doubtful, while test samples that showed S/N above 60% were considered negative.

The true seroprevalence (TP) of anti-SRM antibodies was calculated from the cELISA results using the formula by Greiner and Gardner [19], as follows:

TP = (AP + Sp − 1)/(Se + Sp − 1)(5)

where Se and Sp are the sensitivity and specificity of the cELISA assay used, and AP is the apparent prevalence (AP), calculated by dividing the number of positive samples by the total samples tested.

### 2.3. Determination of the Socio-Economic Impact and Risk Factors for Exposure to SRM

Semi-structured interviews were conducted with individual small ruminant owners and key informants, and focus group discussions (FGDs) with groups of pastoralists. The interactions were in the local language (Ng’akarimojong) and the natural settings of the settlement areas. Probing was done to gain a better understanding of the participants’ descriptions and to limit diversion from relevant topics.

#### 2.3.1. Individual Interviews

Interviews were carried out with 50 individual livestock keepers to collect qualitative and quantitative data on the PPR occurrence, small ruminant management practices, the social and economic impact of the disease, and challenges regarding disease control in individual herds. The interviews were conducted using a semi-structured questionnaire, which was pre-tested with five volunteers whose responses were not included in the final analysis.

#### 2.3.2. Focus Group Discussions

Focus group discussions were conducted with 22 groups each comprising eight to 12 pastoralists in the same settlement areas as the herds where blood samples were collected from the goats and sheep. The discussions followed a prepared checklist and employed participatory epidemiology (PE) tools, including simple ranking, pairwise ranking, proportional piling for morbidity and mortality, and disease impact matrix scoring. The participant groups comprised herdsmen and herd owners and each meeting lasted for about an hour. The discussions were guided by a team of three people: the researcher who led and asked most of the questions, a recorder who wrote down the proceedings, and an interpreter when needed. The team of facilitators was trained first on the application of PE methods and approaches by the researcher, who is a trained participatory disease surveillance (PDS) practitioner. At the end of each day, the appraisal team met to review the recordings and harmonize plans for the following day.

##### Simple Ranking for Common Diseases of Small Ruminants

The participants listed and described the common diseases that affected goats and sheep in their communities. The disease names were in Ng’akarimojong and these were validated by relating them with the clinical signs in the literature. The researcher has experience as a field veterinarian in the Karamoja region and validated as much as possible the participants’ descriptions of the diseases and translated the names to the correct scientific names. The participants ranked the listed diseases in order of their frequency of occurrence in the last year by distributing counters (stones) to each disease. The disease that received the highest number of counters was ranked as the most common in the Karenga district. The top six diseases were recorded for use in the pairwise ranking exercise.

##### Pairwise Ranking for PPR and Other Diseases

The pairwise ranking was conducted as described previously by Dunkle and Mariner [20] to determine the relative importance of the six most important diseases of small ruminants in the Karenga district. For each of the six diseases from the simple ranking exercise, a symbol was drawn next to the local disease name on the flip chart to represent the disease. The recorder drew a six-by-six grid on a flipchart laid on flat ground, with each of the diseases represented on both *x*- and *y*-axes. Each disease was compared individually with all the other diseases one-by-one to determine their relative importance. The comparison was based on social, production, and economic parameters (e.g., cultural value, community interactions, wealth reduction, dowry, milk, weight gain, carcass quality, mortality, marketability, and price from sales). The participants were reminded of what each symbol represented and given time to discuss and reach a consensus. This process was repeated until all disease pairs were compared. For each comparison, the more important disease was recorded in the corresponding grid. The disease that was selected the most was considered most important (rank 1). The reasons for the relative importance of the six diseases were then used as the basis for quantitative matrix scoring of the impact (social, economic, and production) of PPR relative to other diseases.

##### Proportional Piling for Morbidity and Mortality (PPMM)

Proportional piling [20], was used to estimate the morbidity, mortality, recovery, and case fatality rates of the six most important diseases, as determined using simple and pairwise ranking, plus a group of “others” in the herd in the last one year. The participants divided a pile of 100 stones that represented the number of small ruminants in their herd into two piles, to represent the proportions of ill and healthy animals in the past year. The pile representing the ill animals was sub-divided to represent the proportions of illness to each of the top six identified diseases and a group of “others”. Subsequently, the participants divided the pile representing each disease to show the proportions of animals that recovered and those that died from that disease in the past year. The pastoralists, giving reasons for their choice, discussed and agreed on the scores for each disease. The activity was repeated with all the 22 focus groups.

##### Impact Matrix Scoring for Socio-Economic Impact of PPR

The six most important diseases and a group of “others” were included in the matrix to determine the impact of PPR relative to other diseases as described previously [20]. The community-perceived indicators of social and economic impact were those listed during the pairwise comparisons for the diseases as well as the benefits of keeping goats/sheep listed during semi-structured interviews. First, the ten most important impact indicators were selected from the overall list of indicators using simple ranking. This was followed by a proportional piling activity to score the ten selected indicators using 100 counters (stones), which were piled on the indicators according to their relative effect on the livelihood (social, production, economic) in the area. An impact matrix with seven columns and ten rows was then drawn on a flip chart laid on flat ground. The columns were labelled using simple symbols drawn onto pieces of paper to represent the top six diseases and a group of “others”. The indicators and their corresponding number of counters from the proportional piling activity were placed on the *y*-axis of the matrix. The informants divided the counters from each indicator across the diseases to represent the strength of association with each disease.

The facilitators probed the participants to understand the pattern and reasons for the scoring. The participants were given a chance to cross-check and make changes until they all agreed on the scoring. The higher the impact of a disease in relation to an impact indicator, the higher the number of counters allocated to the disease. The matrix scoring was repeated with all the 22 informant groups.

#### 2.3.3. Key Informant Interviews

Fifteen key informants in the Karenga district, selected based on their experience, expertise, and level of interaction with the pastoralist community were interviewed using a semi-structured questionnaire. The informants included *kraal* leaders, local councillors, community animal health workers and field veterinarians. The questionnaire was pre-tested with two key informants to ensure proper understanding, relevance, and logical flow. Information sought during the interviews included small ruminant management and health, outbreaks, and occurrence of PPR, risk factors associated with the spread of PPR, grazing patterns, access to water sources, and prevention and control strategies. Other questions were about challenges regarding controlling the transmission of PPR and the social and economic impacts of the disease in the community.

### 2.4. Data Analysis

Field and laboratory data were managed in Microsoft Excel sheets. Descriptive statistics were used to determine the frequency of responses from key informants, individual farmers, and FGDs. The median and range were determined from the ranking and scoring activities among informant groups. The statistical analysis was conducted using the R Console version 4.0.2, with a confidence interval of 95% and a significance level of 5%. Univariate logistic regression was used to determine the statistical significance of association between the individual animal seropositivity for anti-SRM antibodies and each of the potential risk factors including age, sex, location, animal species, herd structure, herd/flock size, and production system. Significant variables from the univariate analysis (with *p* < 0.05) were analysed in multivariable generalized linear models. First, inter-cluster heterogeneity was estimated by evaluating the null model (without fixed effects and an estimate of *kraal* as a random effect). This was to determine if there was an interdependence of exposure to SRM within a *kraal*. The log-likelihood ratio test was performed to compare the null model and two other models, with or without *kraal* as a random effect. A classical multivariable logistic regression was chosen, employing a forward and backward stepwise Akaike information criteria (AIC) to identify significant risk factors for SRM seropositivity in the Karenga district. The final model was tested for goodness-of-fit using the Hosmer Lemeshow test. The level of agreement among informant groups from the impact matrix scoring was assessed using Kendall’s coefficient of concordance *W*.

## 3. Results

### 3.1. Signalment of Goats and Sheep Sampled

A total of 569 goats and 115 sheep were sampled from seven administrative units (six sub-counties and one town council) in Karenga district, Uganda. The goats and sheep were of the indigenous Small East African breed and the Black-headed Persian breed, respectively. Of the 569 goats sampled, a higher proportion were females (446, 78.4%) compared to males (123, 21.6%). Similarly, more female sheep (105, 91.3%) were sampled than males (10, 8.7%). Most of the animals were more than two years of age (n = 423), followed by one to two years old (n = 174), while a few were less than a year old (n = 87).

### 3.2. Observed Clinical Signs in the Herds

Out of the 684 small ruminants selected for the study, 166 (24.3%) showed one or more clinical signs of PPR. The most common signs observed in the different *kraals* included ocular and nasal discharges, oral lesions, and diarrhoea. The frequency of clinical signs varied by location with a range of 7.4 to 43.9%.

### 3.3. Seroprevalence of Anti-SRM Antibodies

Out of a total of 684 goat and sheep serum samples analysed by cELISA, 334 [48.8%, 95% confidence interval (CI): 45.0–52.6] were positive for anti-SRM antibodies. The overall true prevalence in the Karenga district was 51.4%.

### 3.4. Univariate Analysis of Risk Factors

The results of the univariate analysis for anti-SRM antibody prevalence in the Karenga district showed that out of the seven variables studied, six showed a significant association (*p* < 0.05) with SRM seropositivity (Table 1). Antibodies against SRM were detected in goats and sheep in each of the seven administrative units of the Karenga district. Kapedo sub-county had the highest seroprevalence, while the Karenga sub-county had the lowest prevalence. Seroprevalence was higher in sheep than in goats and was higher in females than males. Animals more than two years old were more exposed to SRM than those one to two years old or those less than a year old.

### 3.5. Multivariable Risk Factor Analysis regarding Exposure to SRM

The final multivariable regression model showed goodness-of-fit to the data (Hosmer Lemeshow test, *p* = 0.739) and included three variables (sub-county, animal species and age). The odds of exposure to SRM were highest for animals in Kapedo and Kawalakol compared to animals in other sub-counties, higher for sheep than goats and higher for animals more than two years of age or one to two years old, compared to those less than one year old (Table 2).

### 3.6. Socio-Economic Impact of PPR in Karenga District

#### 3.6.1. Individual Farmer Interviews

Interviews with 50 small ruminant farmers (12 females and 38 males) across 22 homesteads (*manyattas*) showed that goat and sheep management practices were similar in all locations. The small ruminants were kept in enclosures or *kraals* (*nawi*) within the *manyattas*, with a separation from the large animals (cattle and donkeys). Most of the respondents (n = 47/50, 94%) reported that they kept kids and lambs separate from the older goats/sheep, and those less than a year were herded separately from the rest of the age groups.

Grazing and watering resource management were communal in all 22 locations with one water source shared by five to eight villages. However, all the water sources were temporary, drying up during the dry season and farmers had to move their animals from their villages to other villages, sometimes going as far as the Kaabong (~72 km) and Kitgum (~45 km) districts. At times, farmers used community boreholes that were also unreliable as they often broke down and it took months or years before they got repaired, often through intervention by a non-governmental organization (NGO) or occasionally the government.

The respondents indicated that PPR was one of the most common diseases affecting goats and sheep in their area, and all of them (100%) correctly described the clinical signs associated with the disease. However, only three respondents (6%) had knowledge of PPR management and control, an understanding stemming from their previous training as CAHWs. Even though the remaining 94% indicated no knowledge of management, the majority of the respondents (72%) indicated that they administered supportive treatment to their sick animals by themselves through trial and error, while 24% utilized services of CAHWs, and only 4% had access to services of qualified veterinarians or para-veterinarians. The cost of supportive treatment/management of one PPR case ranged between 5000 and 20,000 Uganda shillings (UGX) (~1.4 and 5.6 USD), not considering the cost of transportation to and from Kaabong (~72 km) or Orom (~45 km) to purchase the supportive drugs, as there were no veterinary drug shops in Karenga district. All the respondents indicated that they isolated sick animals. However, this was only done for animals that presented with obvious clinical signs. All respondents indicated that there had not been any vaccinations against PPR or any other disease of small ruminants, in the Karenga district for the last three years.

All respondents engaged in trade in live small ruminants. For most farmers (82%), goats and sheep were sold to meet immediate household needs, while a few (18%) engaged in small ruminant trade as a business and major source of income. There was no dedicated livestock market in Karenga district, but farmers sold their animals at the trading centres of their respective sub-counties (38%), took to Komuria livestock market (~62 km) in Kaabong district or Orom (~45 km) in Kitgum district (56%), while others sold their animals as far as Narus in South Sudan (~220 km) (6%).

The price of a healthy young goat/sheep (less than six months old) ranged from 20,000 to 45,000 UGX (~5.6 to 12.6 USD), while that of a healthy adult goat/sheep ranged from 80,000 to 130,000 UGX (~22.4 to 36.4 USD). All respondents indicated that PPR reduced the marketability of their animals. In most cases, animals presenting with signs of PPR could not be sold. However, on rare occasions, a sick adult goat or sheep was sold at a lower price of between 35,000 and 50,000 UGX (9.8 and 14.0 USD), a price drop of between 56.3% and 61.5%. All respondents indicated that animals that were taken to the market but did not get sold were returned to the herd/flock, with no quarantine observed.

Most of the respondents (88%) indicated that their goat herds and/or sheep flocks often interacted with wildlife from Kidepo Valley National Park and thought contact of their livestock with wildlife exposed them to PPR. The respondents also associated the occurrence of PPR with the onset of the rainy season when the herders returned from different locations, where they had migrated in search of water and pastures.

#### 3.6.2. Focus Group Discussions

##### Farmers’ Understanding of Diseases of Goats and Sheep

Focus group discussions showed that farmers’ knowledge of the diseases, their clinical signs, and post-mortem presentations aligned to what is described in the scientific literature on small ruminant diseases. Names of diseases in the local Ng’akarimojong language were derived from observed clinical manifestations, post-mortem lesions, or what the farmers perceived as being the cause or transmission agent: for example, PPR was named *louruton* (meaning “with watery stool”) because of its association with profuse diarrhoea that does not respond to known remedies for diarrhoea. Contagious caprine pleuropneumonia (CCPP) was known as *loukoi* (meaning “of the lungs”) because of the effects associated with the lungs. Anaplasmosis was named *lopid* (meaning “of gall bladder”) due to the enlargement of the gall bladder seen at post-mortem, while trypanosomiasis was named *ediit*, meaning “tsetse fly”, because of its transmission cycle that involves tsetse flies.

##### Pairwise Ranking of PPR Relative to Other Diseases

Pairwise ranking of small ruminant diseases by participants in the 22 focus groups showed that CCPP, anaplasmosis, PPR, trypanosomiasis, and helminthosis were the five most important diseases of small ruminants in the Karenga district, in descending order (Table 3). The diseases were prioritized and ranked based on the extent of morbidity, case fatality rates, related veterinary costs, and farmers’ knowledge of management. Kendall’s coefficient of concordance (*W*) indicated a strong agreement between the groups (*W* = 0.62, *p* < 0.001).

##### Disease Incidence and Fatalities

Proportional piling for annual morbidity and mortality due to small ruminant diseases revealed an overall median proportion of illness of 69.5% (range: 58–81%) in the Karenga district. Peste des petits ruminants was scored the second most important cause of morbidity and mortality after CCPP followed by anaplasmosis, helminthosis, trypanosomiasis, mange, and “other” diseases in that order. The case fatality rate was highest for PPR, followed by CCPP and anaplasmosis and the least was for “other” diseases (Table 4)

##### Impact Matrix Scoring of the Socio-Economic Impacts of PPR in Relation to Other Diseases

Participants in the focus group discussions identified several socio-economic impacts of small ruminant diseases in their communities. The median scores of the impact of the top six small ruminant diseases in Karenga district in relation to various socio-economic indicators are shown in Table 5. The impact of the diseases was attributed mainly to the high cost of treatment, high stock mortality, diminished marketability of sick animals, and cause of conflict in the community. Peste des petits ruminants was identified as the biggest cause of conflicts, as it is transmitted through sharing of resources such as drinking points and grazing lands. It was second to CCPP as the disease that resulted in the greatest losses and was associated with high mortality and low marketability of sick animals. It was ranked fourth as a cause of high veterinary cost among goats and sheep in Karenga, after CCPP, trypanosomiasis, and anaplasmosis. Peste des petits ruminants was also identified as being responsible for the loss of farmer status in the community, second to CCPP.

Other socio-economic indicators, including reduced milk production and reduced reproduction capacity of the affected animals, were scored equally for PPR, CCPP, trypanosomiasis, and anaplasmosis. Animals that suffered from PPR were not suitable for paying bride price or for use in rituals (*Nakiriket*). Kendall’s coefficient of concordance (*W*) showed strong agreement (*W* > 0.38, *p* < 0.01) among the groups for all the socio-economic impacts scored, except for “animals not suitable for paying bride price” with a moderate agreement (*W* = 0.26–0.38, *p* < 0.05).

##### Participant Options for PPR Control

The FGD participants proposed solutions to disease control in the area, including effective animal health needs assessment during planning and budgeting at the local government level, prioritization of training and deployment of CAHWs, establishment and support for community-based drug shops by the NGOs, and regular sensitisation of the pastoralists on early warning for disease outbreaks.

#### 3.6.3. Key Informant Interviews

A total of 15 key informants were interviewed, including the only one government animal health officer, one private animal health practitioner, five local councillors, two animal health staff working for NGOs operating in Karenga district and six CAHWs. All the respondents acknowledged the presence of PPR in the Karenga district and mentioned that it is one of the most important diseases of small ruminants, associated with abortions, loss of weight and massive deaths. These losses resulted in significant economic and social impacts including loss of income by farmers due to reduced marketability of the sick animals (n = 15), increasing conflicts between communities that share grazing and watering points (n = 9), increased household poverty, and inability to meet household needs (n = 15). Five of the respondents encountered outbreaks of PPR in the last year. The informants indicated the disease occurred sporadically in different months of the year and different locations, with most reports coming from Kapedo, Kawalakol and Sangar sub-counties. However, the respondents could not recall the number of herds affected or the number of animals lost. All the respondents indicated that control of PPR was a responsibility of multiple stakeholders, with the government taking the lead. In the Karenga district, the last vaccination against PPR was conducted three years before our field visit (2017).

The key challenges in controlling PPR highlighted by the respondents included low technical capacity for surveillance, investigation, and reporting of occurrence due to understaffing of the veterinary department, few trained CAHWs within the district and inadequate capacity building opportunities for the existing animal health personnel. They also noted that there were low incentives to attract private veterinary practitioners to bridge the service delivery gap. There was delayed or sometimes no reporting of PPR outbreaks by farmers as most had limited access to veterinary personnel, and this resulted in untimely implementation of control measures, hence facilitating the continuous spread of the disease in the district.

Cultural practices including migrations, animal raids, and communal grazing increased the risk of spreading PPR and undermined existing efforts put in place to control the disease. This was worsened by the existence of porous borders between Uganda, Kenya, and South Sudan that encouraged uncontrolled animal movements across borders and with it, importation of transboundary diseases such as PPR. It was noted that the Karenga district had infrastructural challenges including lack of quarantine centres for PPR suspects, a poor road network that affected service delivery, and inadequate cold chain facilities for vaccine storage. Moreover, lack of coordination among different stakeholders in animal health resulted in the duplication of interventions and wastage of resources, which could be put in addressing some of the challenges.

## 4. Discussion

The overall true prevalence of anti-SRM antibodies in Karenga district was high (51.4%), and this was in agreement with the 51.4% reported in the northern parts of Karamoja (Kaabong; shares border with Karenga) but higher than the 40.7% reported in the southern parts by Nkamwesiga, Coffin-Schmitt, Ochwo, Mwiine, Palopoli, Ndekezi, Isingoma, Nantima, Nsamba and Adiba [11]. Our findings are also similar to those of Mulindwa, Ruhweza, Ayebazibwe, Mwiine, Muhanguzi and Olaho-Mukani [10], who reported an overall prevalence of 57.6% in four districts of Karamoja: Moroto (south) = 63.2%, Nakapiripirit (south) = 72%, Abim (west) = 1.6%, and Kotido (north) = 85.2%, using a cELISA.

These findings imply that there is a widespread occurrence of PPR among small ruminants in Karamoja, but the exposure to infection is higher in northern than in southern Karamoja. The seroprevalences in this study and previous studies in Karamoja are much higher compared to those observed in other parts of Uganda [21,22]. Aguilar, Mahapatra, Begovoeva, Kalema-Zikusoka, Driciru, Ayebazibwe, Adwok, Kock, Lukusa and Muro [21] reported a seroprevalence of only 1.9% in goats, 5.7% in sheep, and 6.5% in cattle in communities (Buliisa, Kasese, Rubirizi, and Kisoro districts) around Murchison Falls National Park, Mgahinga Gorilla National Park and Queen Elizabeth National Park in western Uganda, while Ruhweza, Ayebazibwe, Mwiine, Muhanguzi, Mulindwa and Olaho-Mukani [22] reported an average seroprevalence of 9.4% in goats and sheep in districts surrounding Karamoja (Soroti, Kumi, Kapchorwa, Katakwi, Sironko, Bukedea, Bukwo, Kaberamaido, Lira, Pader, and Kitgum) in eastern and northern Uganda using a cELISA. This demonstrates different PPR epidemiological situations across the country and the need to design and prioritize control measures based on the context. Elsewhere in eastern Africa, the prevalence of antibodies against SRM were 21.2% in goats in Marakwet East in Kenya and 48.4% in goats and sheep in the Oromia region in Ethiopia, using a cELISA [23].

The practice of moving animals from one location to another (transhumance), as well as sharing grazing areas and watering points, which characterise the production system in the Karamoja region, and as highlighted by the respondents in Karenga district, increases the chances of contact with infected goats and sheep and could facilitate the transmission of SRM. The higher prevalence in the northern Karamoja (Karenga inclusive) can be attributed to the larger small ruminant population size in the north compared to the south [9]. This increases the risk of transmission of PPR among animals, attributed to overcrowding and close contact between infected and susceptible animals.

The cELISA used to detect anti-SRM antibodies in this study does not distinguish between antibodies due to vaccination and natural infection. Given that none of the animals sampled in this study had a history of vaccination against PPR and the study did not include any animal less than six months old, the anti-SRM antibodies detected in this study were due to natural infection with SRM circulating in the communities in and around Karenga district. Antibodies to SRM were detected in all sub-counties in Karenga, which points to the endemicity of PPR in the study area.

Seroprevalence of anti-SRM antibodies varied significantly by location, with higher rates observed in locations that share a border with Kaabong district (internal) and South Sudan, i.e., Kapedo (87.8%), Kawalakol (73.6%), and Sangar (63%), attributed to frequent transboundary movement of livestock across borders for marketing and in search for water and pastures. Locations with a lower prevalence, including the Karenga sub-county (26.8%) and Karenga town council (28.7%), are more inland with fewer cross-border movements or border districts like Kitgum district (internal) where livestock rearing is not a major activity and PPR is not a big concern. The seroprevalence rates are in agreement with the results of clinical examination as a relatively high proportion of animals in Kapedo and Kawalakol sub-counties showed clinical signs (43.9% and 42.9% respectively), which was suggestive of an ongoing outbreak of PPR in these areas, while samples from locations with a lower prevalence were obtained mostly from apparently healthy goats and sheep.

In this study, the prevalence of anti-SRM virus antibodies was higher in sheep (73.9%) than goats (43.8%), although the difference was not statistically significant (*p* = 0.08). Higher prevalence in sheep than in goats has also been reported in previous studies: Woma, et al. [24] in Nigeria (sheep = 23.9%; goats = 22.9%) and Gari, Serda, Negesa, Lemma and Asgedom [23] in the Oromia region, Ethiopia (sheep = 50.9%, goats = 46.7%), using a cELISA. The opposite was true in other studies: Kgotlele, et al. [25] in Ngorongoro, Tanzania (goats = 75.7%, sheep = 42%) and Kihu, et al. [26] in Turkana, Kenya (goats = 40%, sheep = 32%) using a cELISA. However, in all these studies the differences in the seroprevalence of PPR between sheep and goats were statistically insignificant. The higher seroprevalence of PPR in sheep in the present study could be attributed to the higher survival rate in sheep than in goats, as lower morbidity and mortality due to PPR have been reported in sheep than goats by Abd El-Rahim, et al. [27]. Therefore, most goats infected with SRM die before they are examined, while most of the sheep survive and the higher recovery rate translates into detectable levels of anti-SRM antibodies.

Animals more than two years of age had the highest prevalence of anti-SRM antibodies (65.5%) followed by those between one to two years old (24.7%), while small ruminants less than a year old had the lowest seroprevalence (16.1%), suggesting that susceptibility and exposure to PPR increase with age, as supported by Herzog, de Glanville, Willett, Kibona, Cattadori, Kapur, Hudson, Buza, Cleaveland and Bjørnstad [2] and Torsson, et al. [28].

Results from participatory visual techniques showed that PPR was second after CCPP in terms of high morbidity, “high mortality rate”, “low marketability”, and “increased conflicts”. Farmers also identified PPR as the disease with the highest case fatality rate (median 78%). This is consistent with what has been reported in other literature [29,30]. Moreover, the farmers highlighted the loss of status in the community due to high PPR case fatality rates in the Karenga district. In the Karimojong culture, prestige and status are tagged to the number of animals one owns and this comes with power and influence. However, that status is lost when the number of animals is reduced, and animal diseases such as PPR contribute significantly to the loss of status in the community [12,31].

The high mortality rate observed during impact matrix scoring and proportion piling could be as a result of delayed diagnosis, inadequate or delayed supportive treatment. This was supported by the responses during individual farmer interviews, in which 72% of the farmers had limited access to veterinary drugs or professional veterinary services. There was not a single veterinary drug shop in the Karenga district, which prompted farmers to travel to Kaabong, Kotido, or Kitgum (between 45 and 140 km) to buy drugs, which in most cases are also not available in those drug shops. A study in Tanzania study attributed high transmission risks and high prevalence of PPR in Tanzania to differential opportunities in accessing veterinary services, vaccinations, and educational programs [2].

Farmers mentioned that PPR was one of the diseases that rendered animals unsuitable for use in rituals or payment of bride price because it presents with obvious signs such as diarrhoea, oral lesions, and mucopurulent nasal discharges [32,33]. Rituals (*Nakiriket*) and payment of bride price are key aspects of the Karimojong culture that require sacrifice and exchange of livestock, including goats and sheep. However, such animals are required to be in good health and should not have any obvious signs of illness.

Economic losses arising from PPR in Karenga included reduced marketability of the infected animals, high veterinary costs, reduced milk production, and low reproduction, These aspects are similar to those highlighted in the Turkana community in Kenya by Kihu, et al. [34]. During the focus group discussions, the participants mentioned that milk in Karamoja was a major source of nutrition, especially for the herd boys as they move with the animals to far-off grazing areas and therefore diseases such as PPR that lead to a reduction in milk production affect the health of these herders and predispose them to malnutrition as in many instances, they have no alternative source of food. Additionally, dairy production is a target enterprise to improve household income and malnutrition in Karamoja by NGOs and the government, and the high prevalence of PPR threatens the success of such initiatives. Records of reduced reproduction rate, as mentioned by the participants, are consistent with those of Parida, Muniraju, Mahapatra, Muthuchelvan, Buczkowski and Banyard [32] who highlighted PPR as one of the causes of abortion in goats and sheep.

The participants’ report that PPR reduces the marketability of the animals is a valid claim as the disease is characterised by obvious clinical signs in goats and sheep and being a notifiable disease, outbreaks require swift and drastic control measures including the implementation of quarantine. This is a cause for concern for livestock traders because under such circumstances they lack animals to buy. It is also detrimental to the livestock keepers because they would have nowhere to sell such animals, or at best, some livestock traders will buy the animals illegally but at much-reduced prices.

As highlighted by the participants in this study, the relative morbidity due to PPR in Karenga was 14% (range 4 to 27%). As any other livestock-keeping community, the pastoralists are very concerned about the health of their animals and will do whatever it takes to help the animals recover. To the Karimojong, it is difficult to find the resources to facilitate supportive treatment for 14% of a herd affected by PPR. Over 50% of the population in Karamoja live on less than a dollar per day [12,35]. The participants highlighted that they spent between 5000 and 25,000 UGX (~1.4 and 7.0 USD) to manage one PPR case, not including transport costs. This cost is almost 31.3% of the value of the animal, as the price of a healthy adult goat/sheep in Karenga ranged from 80,000–130,000 UGX (22.4 to 36.4 USD).

The participants (during FGD) also highlighted that conflicts, both at household and community levels, were the other impacts of PPR. Peste des petits ruminants was highlighted as a major cause of loss of status and loss of market for the livestock, and 82% of the respondents indicated that they depended on the sale of small ruminants to meet immediate household needs, for instance buying food, paying school fees, and paying medical bills, among others. Conflicts may arise in the home when the family head fails to meet these needs, as highlighted in the report by UNDP [12]. Conflicts at a community level may arise mainly due to the highly contagious nature of the disease. Peste des petits ruminants, being a transboundary disease, spreads easily through shared drinking points and grazing areas [36] and might cause conflict between *kraals*, villages, districts, or even countries because herds from an infected area can be denied access to water and pastures in PPR-free communities. An interesting view raised by the farmers highlighted another form of conflict in the form of livestock raids by communities whose livestock numbers had been reduced as a result of the high mortality rates associated with PPR. To replace their stock, the deprived communities usually resort to raiding animals from other communities. This is a common occurrence in Karenga and other districts in Karamoja and is characterised by armed raids of *kraals*, which are often fatal for both humans and animals.

Farmers’ understanding of the diseases of goats and sheep can be confused in many parts of the world, and if this happens, it can affect the quality of data collected during ranking and scoring. However, in the present study, the within method (probing) and across method (clinical examinations, serology, key informant interviews, FGDs) triangulation has demonstrated significant exposure of small ruminants to PPR and substantial socio-economic impact of the disease in Karenga district. There was consistency regarding the responses from focus group discussions, key informant interviews, and individual small ruminant holders, as well as findings from clinical examinations and laboratory analysis of serum samples. This further demonstrates the relevance of participatory approaches and methods to improve the understanding of animal health problems, disease control options, and the engagement of the communities in the development of solutions for service delivery, control, and surveillance.

## 5. Conclusions

In this study, the true seroprevalence of SRM in the Karenga district was relatively high (51.4%) and the risk factors for exposure were geographical location, animal species, and age. The detection of antibodies against SRM in all administrative units in the district points to a widespread occurrence and endemic status of the disease in the district. Peste des petits ruminants was the second most important disease of goats and sheep after CCPP and was associated with high morbidity and mortality rates, high treatment costs, reduced prices and/or marketability, and affected the cultural value of small ruminants. This impacts food security and livelihood development of the Karimojong pastoralists.

## 6. Recommendations

Given the unique climatic, geographical, and cultural characteristics of the Karamoja region, unique approaches to sustainable disease control are needed. The significance of pastoralists’ situated knowledge has been demonstrated, and this should be tapped into to support the design of better animal health service delivery systems and the development of more effective surveillance and control strategies. In the Karamoja region, the control of animal movements may be difficult due to spatial and temporal variation in the distribution of water and pastures for the animals, and therefore vaccination will be crucial for the control of PPR. Control of PPR should be linked to other vaccinations, especially CCPP, to ensure a wider impact on the public, as well as capacity building of public, private, and community-based entities for vaccine delivery, maintenance of quality monitoring, and record keeping. The long-term impact of vaccination needs to bring everyone (public, private, community) on board and allow effective communication with each other; everyone has their interests and therefore there is a need to come up with a common objective to save time and cost. Peste des petits ruminants is a public good, and therefore continuous external support is required to facilitate within and across border surveillance and vaccination. However, the feasibility of a cost-recovery system, access to vaccines and knowledge should be exploited through, for example, groups or associations of livestock keepers. Surveillance among wildlife is necessary to monitor the maintenance of the virus and infection of wild animals. Further research to establish the circulation of SRM in wild animal populations in Kidepo Valley National Park and other game reserves in the region is required to establish the role of wildlife in the transmission dynamics of SRM.

## Figures and Tables

**Table 1:** Results of univariate logistic regression analysis of potential risk factors for small ruminant morbillivirus (SRM) seropositivity in goats and sheep in Karenga district, Uganda, determined using the competitive enzyme-linked immunosorbent assay. A total of 684 goats and sheep were sampled from November to December 2020. CI—confidence interval.

Variable	No. of Animals Sampled	No. of Positive Samples	Apparent Prevalence (95% CI)	*p*-Value
**Location**				<0.001 ^a^
Lobalangit	109	51	46.8 (37.2–56.6)	
Karenga Town Council	108	31	28.7 (20.4–38.2)	
Kapedo	82	72	87.8 (78.7–94)	
Kawalakol	91	67	73.6 (63.3–82.3)	
Sangar	81	51	63.0 (51.5–73.4)	
Lokori	116	36	31.0 (22.8–40.3)	
Karenga sub-county	97	26	26.8 (18.3–36.8)	
**Animal species**				0.003 ^a^
Caprine (goats)	569	249	43.8 (39.6–48.0)	
Ovine (sheep)	115	85	73.9 (64.9–81.7)	
**Sex**				<0.001 ^a^
Male	133	36	31.9 (19.7–35.5)	
Female	551	298	54.1 (49.8–58.3)	
**Age**				<0.001 ^a^
Less than 1 year	87	14	16.1 (9.1–25.5)	
1–2 years	174	43	24.7 (18.5–31.8)	
More than 2 years	423	277	65.5 (60.7–70.0)	
**Herd size**				<0.001 ^a^
Small (less than 50)	13	7	53.9 (25.1–80.8)	
Medium (50–100)	287	112	39.0 (33.3–44.9)	
Large (>100)	384	215	56.0 (50.9–61.0)	
**Herd structure**				<0.001 ^a^
Goats only	350	131	37.4 (32.3–42.7)	
Mixed, sheep/goats	334	203	68.9 (55.3–66.1)	
**Production system**				0.45
Settled ^b^	242	121	50.0 (43.5–56.5)	
Transhumant	442	213	48.2 (43.4–53.0)	

^a^ Statistically significant difference between levels of a variable (*p* < 0.05).^b^ A settled system was that in which animals were kept within the boundaries of the village where the owners resided, while a transhumant system was where animals were seasonally moved to other locations outside the village boundaries in search of water and pasture.

**Table 2:** Results of multivariate logistic regression analysis of risk factors associated with small ruminant morbillivirus seropositivity in goats and sheep in Karenga district, Uganda. Analysis was done with a binomial generalized linear model. A total of 684 goats and sheep were sampled from November to December 2020. CI—confidence interval. Ref—reference category.

Variable	Odds Ratio	95% CI	*p*-Value
**Location**			
Karenga sub-county	Ref		
Lobalangit	2.1	1.1–4	0.03
Karenga Town Council	1.1	0.6–2.1	0.83
Kapedo	14.5	6.2–36.8	<0.001
Kawalakol	6.0	2.9–13	<0.001
Sangar	4.0	1.9–8.6	<0.001
Lokori	0.7	0.4–1.3	0.26
**Animal species**			
Caprine (goats)	Ref		
Ovine (sheep)	1.7	0.9–2.9	0.08
**Age**			
Less than 1 year	Ref		
1 to 2 years	1.6	0.8–3.4	0.22
More than 2 years	11.1	5.8–22.5	<0.001

**Table 3:** Pairwise ranking of PPR relative to other small ruminant diseases in Karenga district, Uganda. The number of pastoralist focus group discussions = 22. PPR—peste des petits ruminants, CCPP—contagious caprine pleuropneumonia.

9	Local Name	No. of Groups	Minimum Score	Maximum Score	Median Score	Rank
CCPP	*Loukoi*	22	2	5	4	1
Anaplasmosis	*Lopid*	20	1	5	3.5	2
PPR	*Loleo/Louruton*	19	1	5	3	3
Trypanosomiasis	*Ediit*	12	0	5	3	3
Helminthosis	*Ngikur*	16	0	5	2.5	5
Mange	*Emitina/Angaru*	20	0	4	1	6
Orf	*Ngiborok/Ngitubukae*	1	2	2	1	6
Pink eye	*Edeke angakonyen*	1	1	1	1	6
Heartwater	*Lokou*	2	0	1	0.5	9
Footrot	*Ejota*	15	0	3	0	10
Goat pox	*Etom*	4	0	2	0	10

**Table 4:** Comparison of relative morbidity, relative mortality, and case fatality of PPR with other small ruminant diseases in Karenga district, Uganda. Results from proportional piling activities (each using 100 stones) with 22 pastoralist groups (each 8 to 12 participants) in November/December 2020. CCPP: contagious caprine pleuropneumonia; Anap—anaplasmosis, Tryps—trypanosomiasis, Helm—helminthosis. In brackets are the minimum and maximum scores.

	Median Scores for Each Disease						
	CCPP	Anap	PPR	Tryps	Helm	Mange	Others
Relative morbidity	18 (5, 30)	12 (5, 30)	14 (4, 27)	10 (6, 23)	11 (6, 20)	8 (2, 20)	7 (2, 19)
Relative mortality	16 (4, 24)	8 (4, 24)	9 (4, 27)	5 (3, 16)	7 (4, 18)	3 (2, 12)	2 (0, 11)
Case fatality rate	77 (29, 100)	77 (40, 100)	78 (56, 100)	65 (29, 100)	64 (39, 100)	67 (23, 100)	38 (0, 80)

**Table 5:** Comparison of the socio-economic impacts of PPR in relation to other small ruminant diseases in Karenga district, Uganda. The impact was determined by impact matrix scoring with ten indicators, scored using a total of 100 stones, and then the score of each impact was distributed by the participants across the six most important diseases and “others”. The number of informant groups = 22. Anap—anaplasmosis, CCPP—contagious caprine pleuropneumonia, Helm—helminthosis, Tryps—trypanosomiasis.

Impact Indicator	Median, Minimum and Maximum Impact Scores for Diseases						
	CCPP	Anap.	PPR	Tryps.	Helm.	Mange	Others
High treatment cost (W = 0.75 ***)	6.5 (3, 12)	5 (2, 8)	4 (2, 10)	5.5 (2, 12)	1 (1, 3)	2 (1, 4)	1.5 (1, 3)
High mortality rate (W = 0.87 ***)	5.5 (3, 10)	3 (1, 5)	4 (2, 6)	1 (0, 7)	1 (0, 3)	1 (0, 2)	2 (0, 3)
Low marketability (W = 0.62 ***)	4 (1, 5)	1 (0, 3)	2.5 (1, 8)	2 (1, 4)	1 (0, 3)	2 (1, 4)	1 (0, 2)
Low carcass quality (W = 0.63 ***)	2 (1, 4)	0 (0, 2)	0 (0, 1)	1 (0, 2)	1 (0, 3)	0 (0, 2)	0.5 (0, 2)
Reduced milk yield (W = 0.38 ***)	1 (0, 3)	1 (0, 2)	1 (0, 2)	1 (0, 4)	0 (0, 2)	0 (0, 1)	0 (0, 1)
Increased conflicts (W = 0.71 ***)	2 (1, 3)	0 (0, 1)	2 (0, 4)	0 (0, 1)	1 (0, 3)	1 (0, 2)	0 (0, 2)
Animals not suitable for paying bride price (W = 0.43 ***)	2 (1, 5)	1 (0, 2)	1 (0, 4)	1 (0, 3)	1 (0, 3)	1 (0, 2)	1 (0, 1)
Lowered status in the community (W = 0.73 ***)	2.5 (1, 6)	1 (0, 3)	1 (0, 4)	0 (0, 2)	0 (0, 1)	0 (0, 1)	0.5 (0, 3)
Low reproduction (W = 0.63 ***)	1 (0, 1)	0 (0, 2)	1 (1, 4)	1 (1, 3)	0 (0, 1)	0 (0, 0)	1 (0, 3)
Animals not suitable for rituals (W = 0.36 ***)	1 (0, 2)	0 (0, 2)	0.5 (0, 1)	1 (0, 1)	1 (0, 2)	1 (0, 2)	0 (0, 1)

*** *p* < 0.001. *W* = Kendall’s coefficient of concordance, weak agreement: *W* < 0.26, *p* > 0.05; moderate agreement: *W* = 0.26–0.38, *p* < 0.05; strong agreement: *W* > 0.38, *p* < 0.01.

## Data Availability

The data supporting the findings of this study are available on request from the the corresponding author.
